# Mass mortality of the keratose sponge *Sarcotragus foetidus* in the Aegean Sea (Eastern Mediterranean) correlates with proliferation of *Vibrio* bacteria in the tissues

**DOI:** 10.3389/fmicb.2023.1272733

**Published:** 2023-12-01

**Authors:** Ezgi Dinçtürk, Fikret Öndes, Laia Leria, Manuel Maldonado

**Affiliations:** ^1^Fish Disease and Biotechnology Laboratory, Department of Aquaculture, Faculty of Fisheries, Izmir Katip Celebi University, Izmir, Türkiye; ^2^Fisheries Laboratory, Department of Fisheries and Seafood Processing Technology, Faculty of Fisheries, Izmir Katip Celebi University, Izmir, Türkiye; ^3^Department of Marine Sciences and Applied Biology, Faculty of Science, University of Alicante, Alicante, Spain; ^4^Department of Aquatic Ecology, Centro de Estudios Avanzados de Blanes (CEAB-CSIC), Girona, Spain

**Keywords:** heat waves, invertebrate mortality, ocean warming, *Vibrio fortis*, *Vibrio gigantis*, *Vibrio owensii*, vibriosis

## Abstract

In the last two decades, episodes of mass mortality in benthic communities have often been associated with climatic anomalies, but the ultimate mechanisms through which they lead to death have rarely been identified. This study reports a mass mortality of wild sponges in the Aegean Sea (Turkey, Eastern Mediterranean), which affected the keratose demosponge *Sarcotragus foetidus* in September 2021. We examined the occurrence of thermo-dependent bacteria of the genus *Vibrio* in the sponges, identified through 16S rRNA of colonies isolated from sponge tissue in specific culturing media. Six *Vibrio* sequences were identified from the sponges, three of them being putatively pathogenic (*V. fortis*, *V. owensii*, *V. gigantis*). Importantly, those Vibrios were isolated from only tissues of diseased sponges. In contrast, healthy individuals sampled in both summer and winter led to no *Vibrio* growth in laboratory cultures. A 50 years record of sea surface temperature (SST) data for the study area reveals a progressive increase in temperature from 1970 to 2021, with values above 24°C from May to September 2021, reaching an absolute historical maximum of 28.9°C in August 2021. We hypothesize that such elevated SST values maintained for several months in 2021 promoted proliferation of pathogenic *Vibrio* species (thermo-dependent bacteria) in *S. foetidus*, triggering or aggravating the course of sponge disease. Thus, vibrioisis emerges as one of the putative mechanisms through which global water warming in the Mediterranean Sea translates into sponge mortality. The historical time course of temperature data for the studied area in the Aegean Sea predicts that recurrent waves of elevated SST are likely to occur in the coming summers. If so, recurrent disease may eventually eliminate this abundant sponge from the sublittoral in the midterm, altering the original bathymetric distribution of the species and compromising its ecological role.

## 1 Introduction

Sponges play important functional roles in many marine ecosystems, from creation of habitat to benthic-pelagic coupling of inorganic and organic nutrients in the marine food web (Bell, 2008; Maldonado et al., 2012; de Goeij et al., 2017). Like other sessile benthic groups (e.g., anthozoans, bivalves, ascidians), sponges have been facing recurrent episodes of mass mortality worldwide (Cerrano et al., 2000; Webster, 2007; Stabili et al., 2012; Di Camillo et al., 2013; Carella et al., 2019; Ereskovsky et al., 2019; Luter and Webster, 2022). The multiplication of contemporary studies on this subject could erroneously lead one to think that these mortalities are exclusively a recent phenomenon, but some reports on mass mortality of commercial sponges date back a century (Allemand-Martin, 1914; Galtsoff et al., 1939). Yet, it appears that the periodicity of these mortality episodes has drastically increased over the last two decades, as research compiled from the well-studied Mediterranean Sea points out (Garrabou et al., 2022).

A variety of factors and conditions have been tentatively related to mass sponge mortalities, but the ultimate agents/mechanisms causing mortality have seldom been elucidated. Local accumulation of pollutants, introduction of alien species, ecological imbalances caused by over-harvesting commercial benthic organisms, environmentally-induced dysbiosis of the symbiotic microbiome, infection by pathogens from the water column, and anomalous climate patterns leading to heat waves – among other factors – have been pointed out as general scenarios associated to different cases of sponge mass mortality (Webster, 2007; Stabili et al., 2012; Fortunato et al., 2022; Luter and Webster, 2022; Botté et al., 2023). The latter two (pathogens and water warming) are perceived to be particularly important drivers of sponge mortality, and they can be related to each other through the hypothesis that increasing values of sea surface temperature (SST) in turn increase microbial pathogenicity or sponge susceptibility or both (Vacelet et al., 1994b; Sweet et al., 2015). Under such scenario, the genus of *Vibrio* bacteria emerges as a putative candidate for at least some cases of sponge mortality, a possibility that remains poorly investigated in this animal group. *Vibrio* consists of thermo-dependent bacteria that are often pathogenic or facultative pathogenic to a wide variety of aquatic organisms (corals, bivalves, fish, etc.), and whose niche expansion is being facilitated by the global ocean warming (Frydenborg et al., 2014). While microbial agents, in general, have putatively been blamed as responsible for several episodes of sponge disease (Vacelet et al., 1994a; Maldonado et al., 2010a; Stabili et al., 2012; Sweet et al., 2015), very few studies have been able to unequivocally identify the specific pathogens. Such a situation is not surprising given the complexity of the microbiome of most sponges (Schmitt et al., 2007; Webster and Taylor, 2012). Among the few cases in which the microbial pathogen was identified (Rützler, 1988; Webster et al., 2002; Choudhury et al., 2015; Sweet et al., 2015), no *Vibrio* species was involved, being the etiological agents identified as: (1) *Pseudoalteromona agarivorans* (initially misidentified as *Sulfitobacter pontiacus*), related to both the sponge white patch (SWP) and sponge boring necrosis (SBN), and (2) *Hormoscilla* sp., linked to mangrove sponge disease (MSD). To our knowledge, the occurrence of *Vibrio* spp. in previous sponge diseases has only been tentatively suggested for the sponge *Ircinia fasciculata* (Maldonado et al., 2010a) and experimentally confirmed for the related species *Ircinia variabilis* by Stabili et al. (2012), who cultured *Vibrio rotiferanius* in the laboratory after agar inoculation with tissue of diseased individuals.

Herein we report on a disease that affected the populations of the sponge *Sarcotragus foetidus* Schmidt, 1862 at the coast of Turkey (Aegean Sea) in summer 2021. To our knowledge, this is the first report on massive mortality of wild sponges in the Turkish Aegean Sea. It is worth noting that this episode appears to be connected to the mass mortality of shallow-water, stony corals reported from the North Aegean Sea, also in summer 2021 (Antoniadou et al., 2023). According to long term-records of sea surface temperature (SST), this area of the Eastern Mediterranean has experienced over 35 marine heatwave events during the last decade, being currently considered as a “hot spot” for marine heatwaves (Androulidakis and Krestenitis, 2022; Juza et al., 2022). Surprisingly, despite the prominence of the Aegean Sea as a potential “natural laboratory” to monitor the effects of seawater warming, very few studies on mass mortality of benthic invertebrates have been conducted to date (Garrabou et al., 2022; Antoniadou et al., 2023). In the present study, we have examined basic aspects of the microbiology and histopathology of the disease of the sponge *S. foetidus*, directly focusing on whether proliferation of *Vibrio* spp. was detectable in the tissue of diseased sponges. Our results point to an interesting correlation between the proliferation of some *Vibrio* species in the sponges and the development of disease, providing a possible mechanistic link between water warming and sponge disease, which will hopefully open new avenues of research.

## 2 Materials and methods

### 2.1 Studied species

*Sarcotragus foetidus* – commonly known as dark stinging sponge – is a so-called corneous, horny or keratose sponge, that is, a sponge that lacks a mineral skeleton but has instead developed a skeleton of fibers made out of a collagen-based protein, spongin. It belongs to family Irciniidae of the class of Demospongiae. The species is distributed in the Eastern and Western Mediterranean Sea, also in the North Atlantic Ocean: Cape Verde, The Canary Islands, Madeira, Azores (Cruz, 2002; Pavloudi et al., 2016). The species occurs at diverse habitats, including rocky substrates, coralligenous, and detrital and muddy bottoms, as well as in caves. Its bathymetric distribution ranges from 3 to 400 m (Manconi et al., 2013). Its body is black to dark-grey in colour, a slightly flattened sub-sphere in shape, and can reach up to 1 m in diameter and 50 cm in height.

### 2.2 Study site, sponge density, and disease incidence

**Figure 1:**
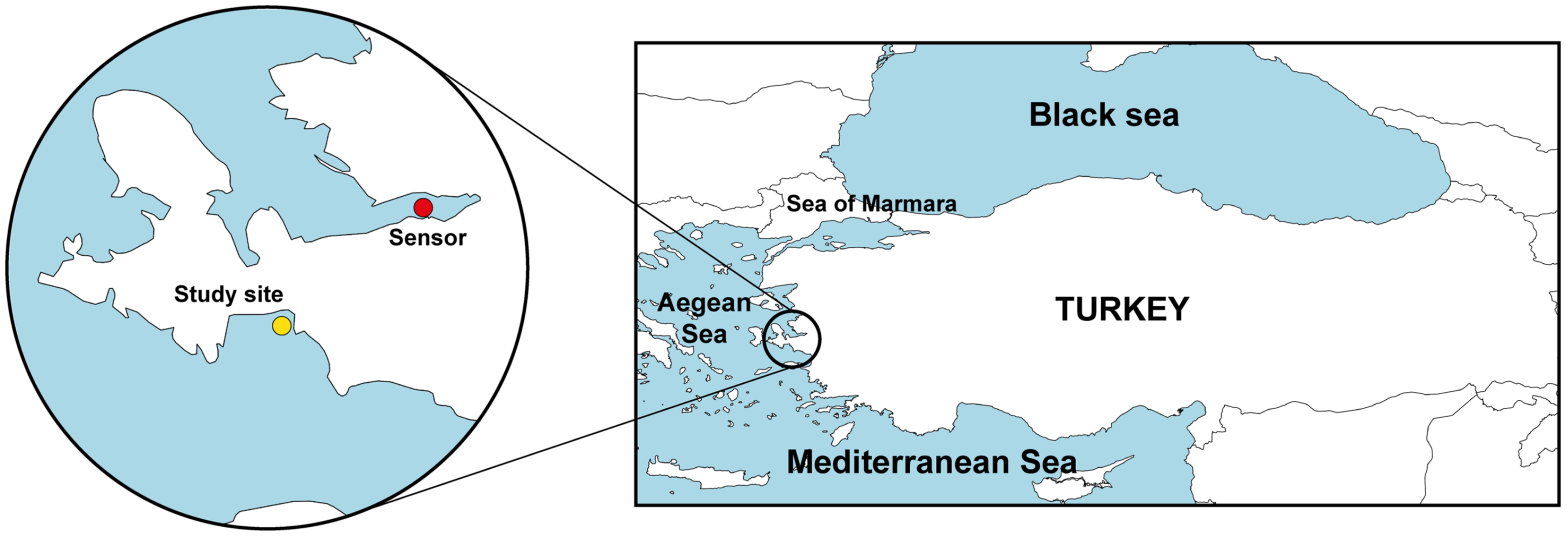
Map showing the location of the study site (Seferihisar, Aegean Sea, Turkey; yellow circle) and the location of station 29 station (Izmir) of the Sea Surface Temperature Network of the Meteorological Service of the Ministry of Agriculture and Forestry of Turkish Republic that provided SST data (red circle).

**Figure 2:**
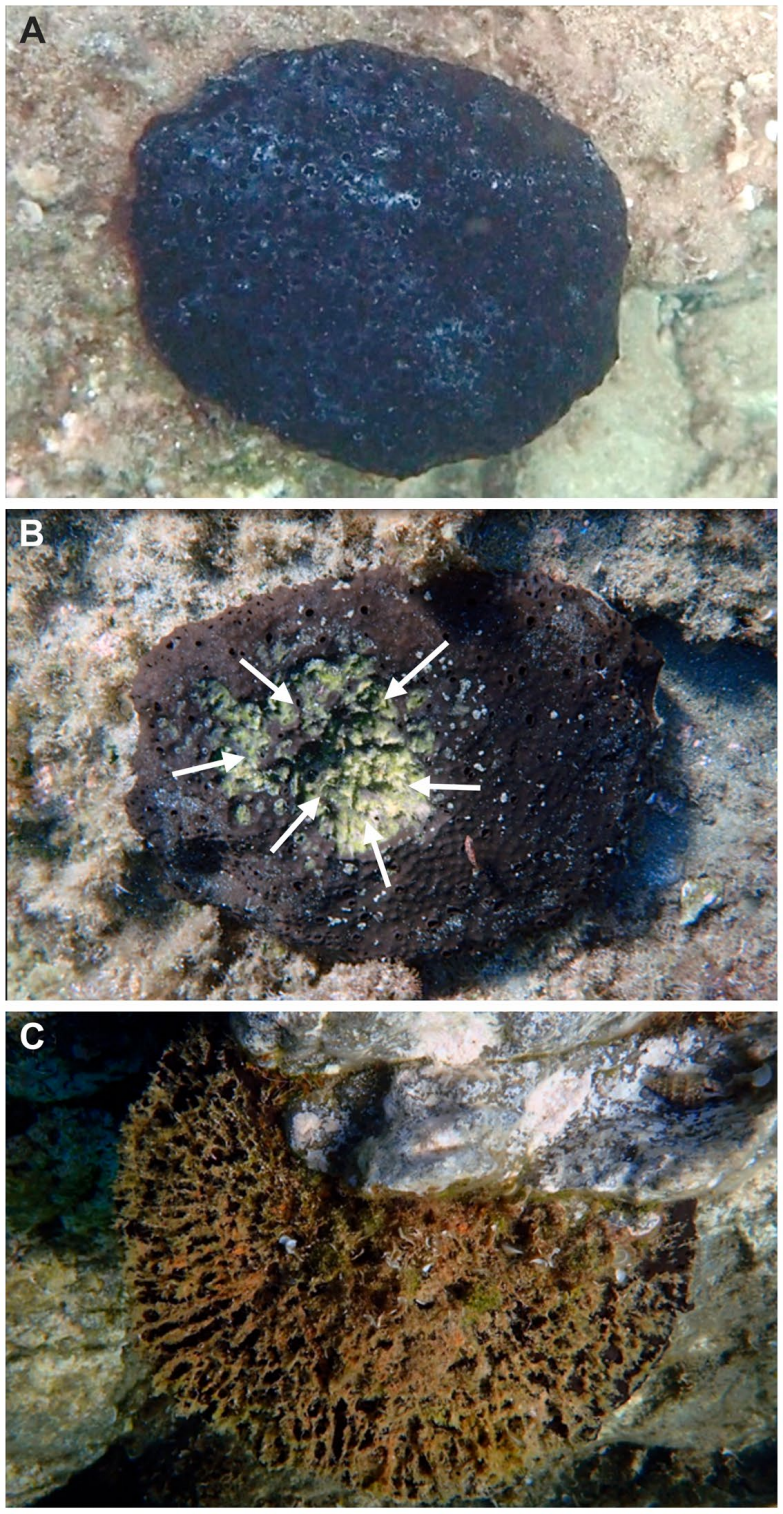
Macroscopic comparative views of disease development in *Sarcotragus foetidus*: **(A)** healthy individual. **(B)** Individual affected by partial necrosis (bleached area marked with arrows). **(C)** Dead individual, in which the sponge tissue has been degraded and the internal fiber skeleton becomes exposed. Photos by Fikret Öndes.

After noticing an epidemic outbreak in August 2021, immediate scuba diving surveys were conducted from 1 to 7 m to assess disease incidence in the studied population (38°11′56”N, 26°46′38″E; Seferihisar, Aegean Sea; Figure 1) and to collect tissue samples. Further dives were conducted in September 2021 to determine sponge density and body size distribution. Quantifications were conducted following the line transect method in randomly selected areas (Plumptre, 2000), with 6 random transects ranging in length from 100 to 350 m, resulting in a total of 5,832 m^2^ examined. A second survey for disease incidence was conducted in December 2021. Throughout the dives, each specimen of *S. foetidus* that was found was photographed using an underwater camera Olympus Tough TG-4 and its health condition registered *in situ* (Figure 2). Macroscopy damage level in the sponges was rated under three categories (1 = healthy individuals, with no macroscopically evident damage at the sponge surface, 2 = individuals with partial necrosis, lesions representing <50% of sponge surface area; and 3 = dead individuals or dying individuals, with lesions extending over >50% of sponge surface). The diameter of each sponge was measured *in situ* using a ruler, and measurement data were linked to the underwater pictures taken for each sponge in order to calibrate digital Images for further processing with ImageJ Software. To examine whether sponge body size (a proxy indicator of relative age) might be implicated in susceptibility to the disease, differences in mean body diameter between healthy and diseased individuals were examined using the Mann–Whitney U test. Statistical analyses were performed using SPSS software (ver. 20).

### 2.3 Seawater temperature

We analyzed a historical series of sea surface temperature (SST) data for a 50 years period between 1970 and 2021. Data were obtained through station 29 (38°24′20”N, 27°04′19″E; Figure 1) of the Sea Surface Temperature Network of the Meteorological Service of the Ministry of Agriculture and Forestry of Turkish Republic: https://www.mgm.gov.tr/eng/marine-sea-surface-temperature.aspx. Data represent monthly averages. From 1970 to 2008 data were obtained by hand thermometers submerged at 2 m depth at 07:00 am. From 2008 to 2021, temperature values were automatically registered from a depth of 2 m using Vaisala QMT103 PT100 sensors.

### 2.4 Microbiological and pathological study

Samples for the microbiological study were collected with scalpels during dives in both summer (9 individuals with incipient signs of disease and 5 healthy individuals) and winter (12 individuals, all healthy since symptomatic individuals did not occur) of 2021. Upon collection, tissue samples from diseased and healthy sponges were carried in separated ice boxes to prevent contamination and taken to Izmir Katip Celebi University Faculty of Fisheries Fish Disease and Biotechnology Laboratory for microbiology, molecular biology and histopathology protocols.

**Figure 3:**
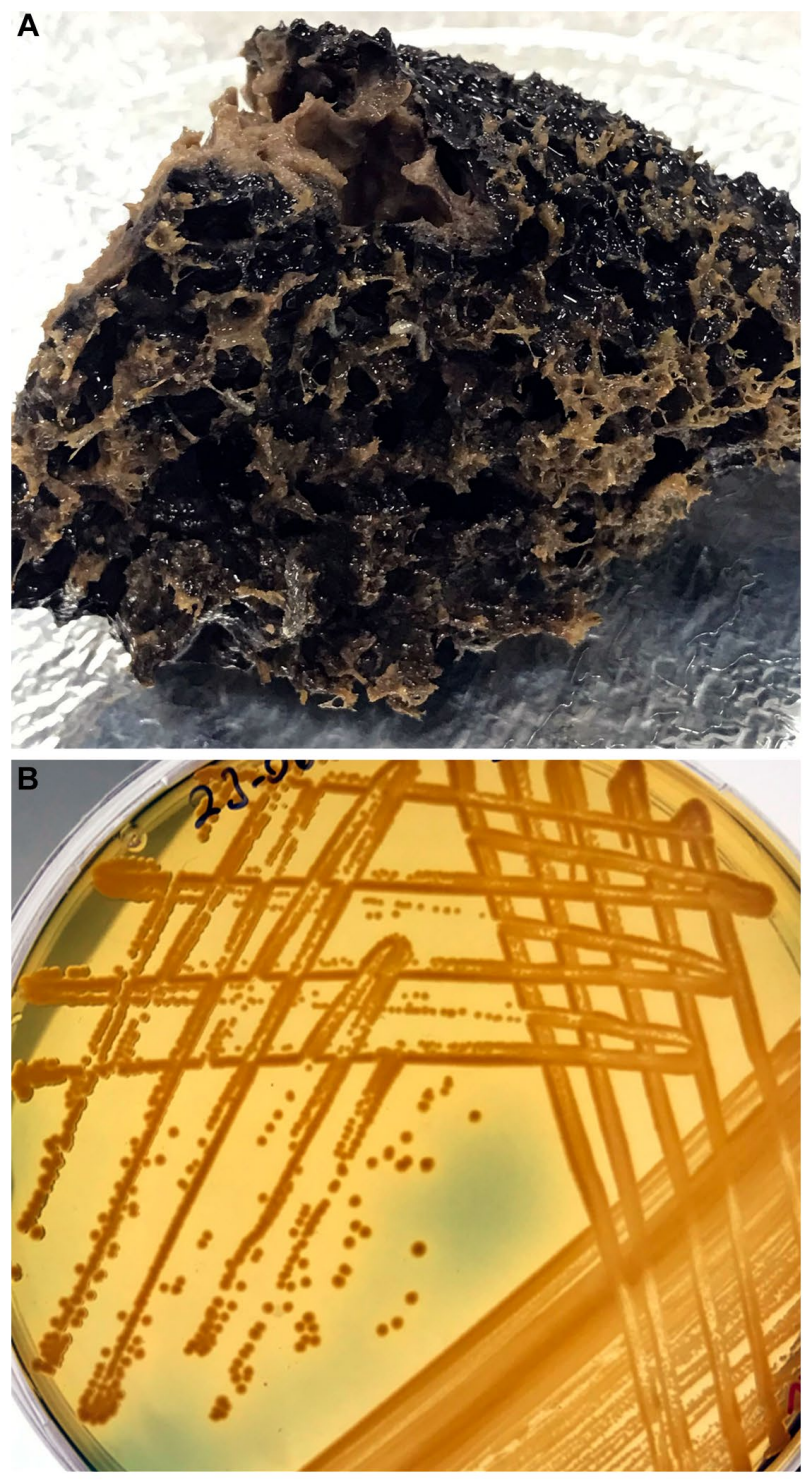
**(A)** Diseased individual of *Sarcotragus foetidus* collected to inoculate culturing media for *Vibrio*. **(B)** View of bacterial growth in Tryptic Soy agar supplemented with 1.5% NaCl, thiosulfate citrate bile salt sucrose (TCBS) agar, and Marine Agar after incubation at 21°C for 48 h.

Tissue samples from both healthy and diseased sponges were first rinsed with sterile seawater to remove loosely associated organisms, then cut into small pieces (approximately 1 cm^2^) using sterile scalpel blades. Bacteria isolation followed the protocol by Stabili et al. (2012). After sonicating the tissue, each sample was plated onto a culturing medium selective for *Vibrio* spp., consisting of Tryptic Soy Agar (TSA) supplemented with 1.5% NaCl, Marine Agar (Merck, Germany), and thiosulfate citrate bile salt sucrose (TCBS) agar. Plates were incubated at 21°C for 48 h (Figure 3). The putatively pure colonies were streaked onto medium and a preliminary characterization was performed through colony morphology, Gram staining, sensitivity to the Vibriostatic O/129 agent, and oxidase/catalase assays, according to standard methods (Cappuccino and Welsh, 2019).

Amplification of 16S rRNA gene was conducted for molecular identification of the isolated colonies. For DNA isolation, EurX GeneMATRIX Bacterial & Yeast DNA Isolation Kit (EurX Molecular Biology Products, Poland) was used. The quality and density of the DNA were measured with Thermo Scientific Nanodrop 2000 (United States). PCR amplifications were performed using universal primers 27F (5’ AGAGTTTGATCMTGGCTCAG 3′) and 1492R (5’ TACGGYTACCTTGTTACGACTT 3′), following Frank et al. (2008). The PCR reactions were carried out through initial denaturation at 95°C for 5 min, following 40 cycles at 95°C, 45 s for denaturation at 57°C, 45 s for annealing at 72°C, 60 s for extension at 72°C, and 5 min as final extension.

The PCR amplification products were verified with 1.5% agarose gel prepared with 1 X TAE buffer at 100 volts for 90 mins electrophoresis and the band screening was observed in UV light using ethidium bromide dye. The PCR products were purified with the MAGBIO “HighPrep^™^ PCR Clean-up System” (AC-60005) according to the manufacturer’s instructions. The amplified products were sent to Macrogen Netherlands Laboratory for sequence determination with ABI 3730XL Sanger sequencing device (Applied Biosystems, Foster city, CA) and BigDye Terminator V3.1 Cycle Sequencing Kit. CAP contig assembly algorithm was used in BioEdit software to produce a consensus sequence. Sequences have been deposited ine GenBank database and accession numbers are available in Table 1. To build a phylogenetic framework on which classify the extracted 16S rRNA sequences, publicly available 16S rRNA sequences of 162 *Vibrio* species were downloaded from the “List of Prokaryotic names with Standing in Nomenclature” (LPSN) database (Parte et al., 2020; https://lpsn.dsmz.de/genus/vibrio, last accessed on 6/6/23). All sequences were aligned using the MAFFT online software with the Auto strategy (Katoh et al., 2019). Sequence alignment in fasta format is provided as Supplementary Datasheet S1. Phylogenetic trees were inferred using both Maximum Likelihood and Bayesian Inference methods, implemented in the programs IQ-TREE 1.6.0 (Nguyen et al. 2015) and MrBayes 3.2 (Ronquist et al., 2012), respectively. Phylogenetic inference with IQ-TREE was carried out setting 5.000 replicates of ultrafast bootstrap (Hoang et al., 2018) and using the ModelFinder Plus option (Kalyaanamoorthy et al., 2017), which searches and implements the best fitting evolutionary model to the data. The selected model was the General Time Reversible model, with empirical base frequencies plus a four-category FreeRate model (GTR + F + R4). The Bayesian phylogenetic inference was performed with two runs of 2.000.000 generations, four chains, sampling every 200 generations and applying a default burn-in of 25%. The best fitting model among the ones implemented in MrBayes was selected with ModelFinder using IQ-TREE, which resulted in the General time reversible, with empirical frequencies, invariable sites and a four-category gamma distribution model (GTR + F + I + G4). Once the MrBayes analysis was finished, the convergence between the two runs was examined by checking that the standard deviation of the split frequencies was smaller than 0.01. Resulting phylogenies were visualized with FigTree Software^1^ and prepared for final figures with Adobe Illustrator Software.

**Table 1:** Alignment length, number of variable sites, identical sites, and pairwise identity for the *Vibrio* sequences identified from *Sarcotragus foetidus* in the present study (query species) and their closest relatives in each clade.

Clade	Query species (GenBank Accession Number)	Related vibrio species (GenBank Accession Number)	Alignment length (bp)	Variable sites (bp)	Identical sites (bp)	Pairwise identity
1	*Vibrio* sp. (OK036808)	*Vibrio owensii* (MH315816)	1,433	9	1,349	99.3%
2	*Vibrio* sp. (MZ900920)	*Vibrio antiquarius* (AF319769)	1,476	15	1,363	98.9%
2	*Vibrio* sp. (MZ900920)	*Vibrio natriegens* (AB680922)	1,479	19	1,359	98.6%
2	*Vibrio* sp. (MZ900920)	*Vibrio neocaledonicus* (KT989844)	1,474	14	1,363	99.0%
2	*Vibrio* sp. (MZ900920)	*Vibrio chemaguriensis* (MG356329)	1,465	31	1,353	97.8%
3	*Vibrio* sp. (OK037006)	*Vibrio fortis* (AJ514916)	1,505	22	1,400	98.6%
4	*Vibrio* sp. (OK036959)	*Vibrio mytili* (X99761)	1,441	27	1,231	97.9%
5	*Vibrio* sp. (MZ901309)	*Vibrio celticus* (EF599162)	1,546	10	1,362	99.3%
5	*Vibrio* sp. (MZ901309)	*Vibrio* sp. (OK037007)	1,416	8	1,364	99.4%
5	*Vibrio* sp. (MZ901309)	*Vibrio kanaloae* (AJ316193)	1,495	24	1,349	98.4%
5	*Vibrio* sp. (MZ901309)	*Vibrio toranzoniae* (EU541606)	1,454	17	1,355	98.8%
5	*Vibrio* sp. (MZ901309)	*Vibrio artabrorum* (EF599164)	1,544	10	1,363	99.3%
5	*Vibrio* sp. (MZ901309)	*Vibrio coralliirubri* (HG942391)	1,545	5	1,367	99.6%
5	*Vibrio* sp. (MZ901309)	*Vibrio gigantis* (EF094888)	1,498	6	1,366	99.6%
5	*Vibrio* sp. (OK037007)	*Vibrio celticus* (EF599162)	1,546	9	1,409	99.4%
5	*Vibrio* sp. (OK037007)	*Vibrio kanaloae* (AJ316193)	1,496	22	1,396	98.6%
5	*Vibrio* sp. (OK037007)	*Vibrio toranzoniae* (EU541606)	1,455	17	1,400	98.8%
5	*Vibrio* sp. (OK037007)	*Vibrio artabrorum* (EF599164)	1,544	8	1,411	99.5%
5	*Vibrio* sp. (OK037007)	*Vibrio coralliirubri* (HG942391)	1,545	5	1,413	99.6%
5	*Vibrio* sp. (OK037007)	*Vibrio gigantis* (EF094888)	1,498	5	1,413	99.7%

Counts for “alignment length (bp)” include missing data.

Tissue samples collected for histological inspection were prepared according to standard methods for light microscopy, as described by Cerrano et al. (2001). Tissue sections were mounted on slides, stained with Hematoxylin-Eosin (H&E), and examined under a light microscope Olympus CX22RFS1.

## 3 Results

### 3.1 Sponge abundance and disease incidence

The survey of 5,832 m^2^ of bottom revealed high abundance of *S. foetidus*, with a mean (±SD) density of 0.02 ± 0.01 ind. m^−2^, and maximum of up to 4 ind. m^−2^. Typically, individuals were separated from each other by 2 to 4 lineal meters. Regarding the incidence of disease in the population during summertime (August 2021), a total of 64% of the 117 individuals surveyed were healthy (Figure 2A), 27% affected by partial necrosis (Figure 2B), and 9% dying or dead (Figure 2C). Importantly, diseased individuals were not found during winter surveys.

**Figure 4:**
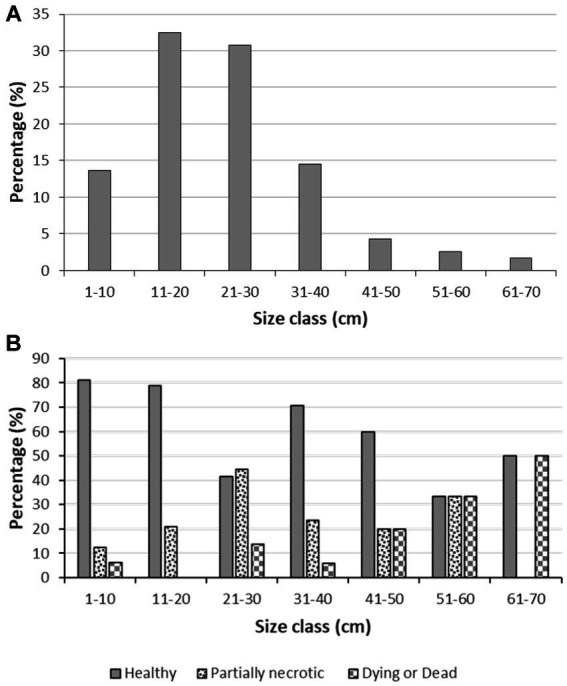
Body size distribution data. **(A)** Body size distribution in the *Sarcotragus foetidus* population, **(B)** Body size distribution of healthy, partially necrotic, and dying or dead individuals of *S. foetidus* (*n* = 117).

The body size of the *S. foetidus* individuals (Figure 4A) ranged in the population from 4 to 70 cm in diameter, with a mean (±SD) value of 23 ± 13 cm. Sponges larger than 40 cm in diameter were rarely observed. The mean size of the healthy sponges was slightly smaller (21 ± 12 cm) than that of the unhealthy individuals (25 ± 11 cm) with statistical significance, according to a Mann–Whitney U test (U = 908.500, *N*_1_ = 75, *N*_2_ = 32, *p* = 0.047). Furthermore, 90% of dying or dead individuals were larger than 20 cm (Figure 4B), a pattern suggesting that the largest individuals – probably also the older ones – may be more susceptible to disease.

### 3.2 Microbiological and molecular findings

**Figure 5:**
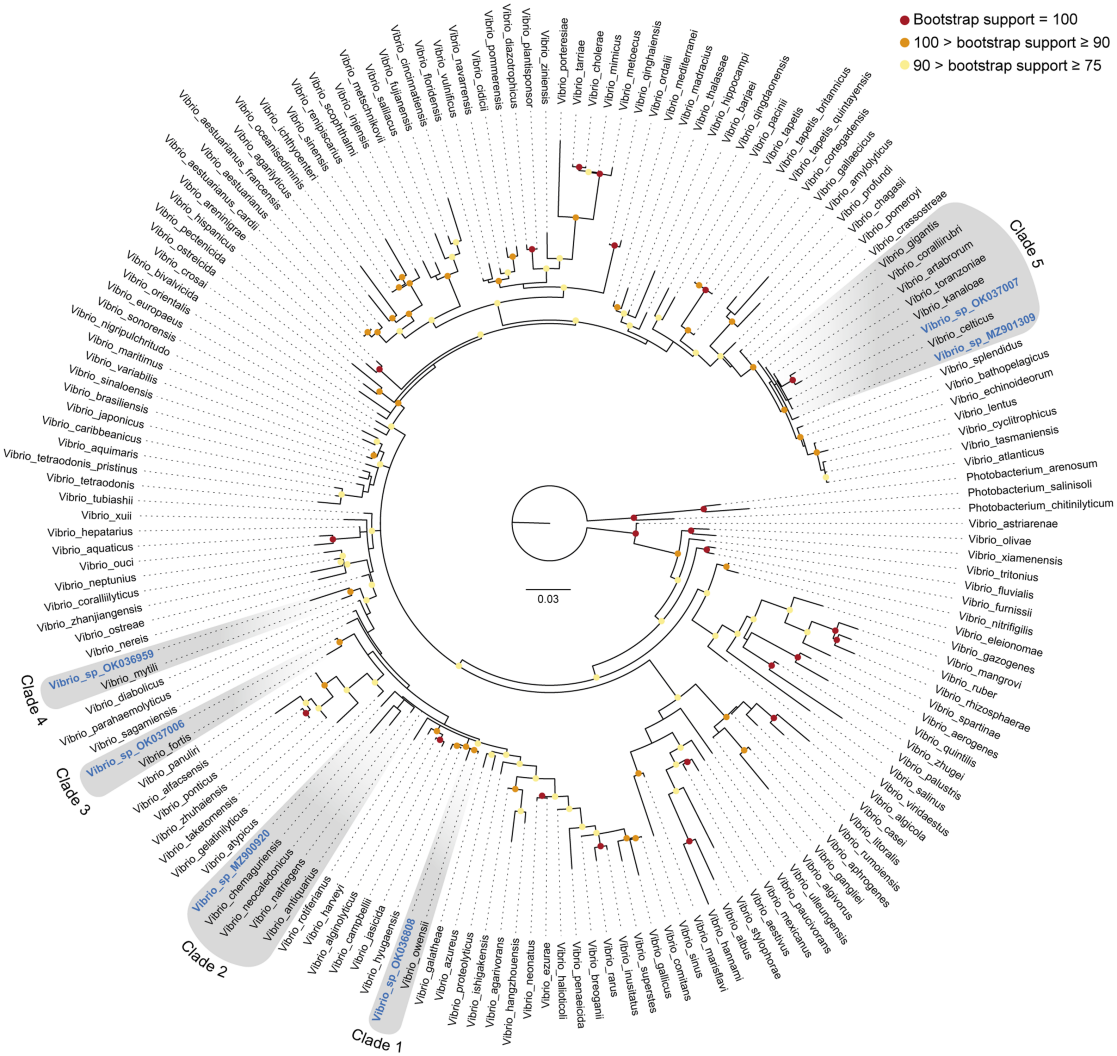
16S rRNA Maximum Likelihood phylogeny of the genus *Vibrio*, including six new sequences identified from tissue of the sponge *Sarcotragus foetidus*. Phylogenetic inference was performed using IQTREE software (see Methods), considering three species of the related genus *Photobacterium* as outgroup. The six new sequences reported in this study are highlighted in blue and become distributed into five different clades (clades 1 to 5, highlighted in grey). Bootstrap support (%) of nodes is indicated with colored circles (red: bootstrap support = 100%; orange: 100% > bootstrap support ≥ 90%; yellow: 90% > bootstrap support ≥ 75%). Nodes without colored circle indicate support values lower than 75%. A maximum likelihood phylogeny providing exact support values for all nodes is given as Supplementary Figure S1. Aligned sequences are available in fasta format as Supplementary Datasheet S1. Accession numbers are given in Supplementary Table S2. Scale bar represents 0.03 nucleotide substitutions per site.

**Figure 6:**
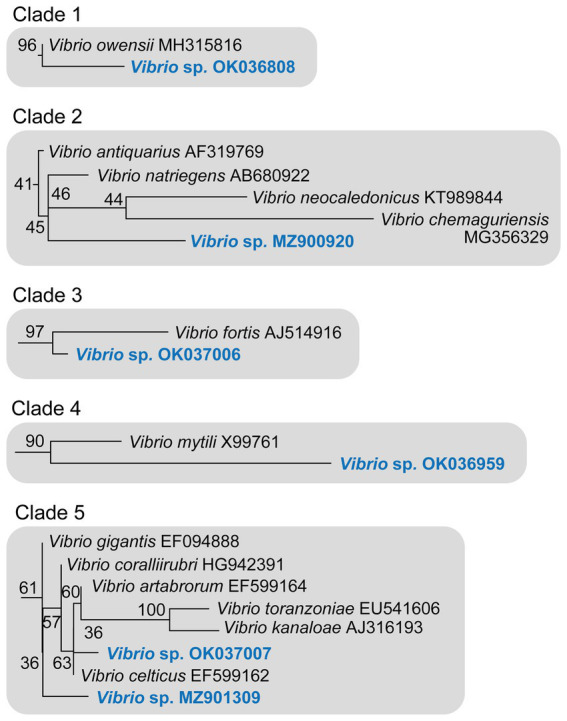
Detail of the five 16S rRNA phylogenetic clades including the six new *Vibrio* sequences identified in *S. foetidus*. The herein identified *Vibrio* sequences are highlighted in blue. Numbers at nodes correspond to the bootstrap support obtained in the maximum likelihood phylogenetic inference (see Figure 5 and Supplementary Figure S1 for a complete tree).

During underwater inspections, the macroscopic signs of disease manifested as bleached, depressed, necrotic areas, sometimes also covered by a whitish biofilm. From these diseased tissue samples, gram-negative, oxidase- and catalase-positive, O/129-sensitive bacteria were isolated, producing yellow colonies on TCBS agar (Figure 3B). When sponge tissues from healthy sponges collected in either summer or winter were cultured, no Gram-negative bacteria sensitive to O/129 were recovered. Sequencing of 16S rRNA extracted from the cultured colonies yielded 6 different sequences (Supplementary Datasheet S1), clustering in five different subclades of the *Vibrio* phylogeny (Figures 5, 6; Supplementary Figure S1). It is worth noting that the resulting phylogeny of *Vibrio*, based on 16S rRNA sequences for a total of 162 species/subspecies (plus our 6 sequences), shows many nodes with low (i.e., statistically unreliable) support (Figure 5; Supplementary Figure S1). It reflects that as the number of described species in *Vibrio* has risen, the resolution power of the 16S marker have notably decreased, because it is characterized by large invariant regions and very high percentage identity between related species (Table 1). A consequence of the lack of resolution of the 16S rRNA is also that the different runs of the Bayesian phylogenetic inference did not converge to a result, which is the reason why we are only providing results of maximum likelihood phylogenetic inference. Nevertheless, despite the general lack of phylogenetic resolution provided by the 16S rRNA marker, the obtained results do allow us to tentatively identify the isolated *Vibrio* species, though with caution (see Discussion: “Taxonomic interpretation of 16S rRNA Vibrio sequences”).

In this context, sequence MZ900920 obtained from *S. foetidus* cultures represents an undescribed sequence of *Vibrio*, which probably belongs to a non-pathogenic species, because it is highly related (>98.7% pairwise identity) to three presumably non-pathogenic species (clade 2, Figures 5, 6, Table 1): *Vibrio antiquarius* from deep-sea hydrothermal vents (Hasan et al., 2015), *Vibrio neocaledonicus* from New Caledonia, producing an exopolysaccharide highly corrosive to metals (Chalkiadakis et al., 2013), and *Vibrio natriegens*, a fast growing species used in bioengineering (Weinstock et al., 2016). Likewise, sequence OK036959 appears to represent an undescribed, non-pathogenic species related (97.9% identity) to *Vibrio mytili* (clade 4; Figures 5, 6), which is also reported to be non-pathogenic (Darshanee Ruwandeepika et al., 2012). In contrast, sequences OK036808 and OK037006 belong, respectively, to either strains or undescribed species closely related to *Vibrio owensii* (clade 1; 99.3% identity) and *Vibrio fortis* (clade 3; 98.6% identity). These two species are reported as pathogenic for a wide array of marine organisms (Cano-Gómez et al., 2010; Ushijima et al., 2012; Wang et al., 2016; Liu et al., 2018, 2021). The two remaining sequences, OK037007 and MZ901309, cluster in the Gigantis group (clade 5; Figures 5, 6). According to the phylogenetic analysis, OK037007 sequence is closer to *Vibrio celticus* (94.4% identity) – known to be pathogenic to clams (Beaz-Hidalgo et al., 2010) – but with unreliable node support. However, an examination of the alignment (Table 1, Supplementary Datasheet S1) indicates that the number of nucleotides which differ in the comparison of sequences is 9 versus *V. celticus*, but only 5 versus either *V. coralliirubri* (99.6% identity) and *V. gigantis* (99.7% identity). While *V. coralliirubri* has been reported to date as a non-pathogenic species from the mucus of Western Mediterranean red corals (Poli et al., 2018), *V. gigantis* is commonly associated with farms of seabass farms and invertebrate aquaculture in the Eastern Mediterranean (Beleneva and Kukhlevskii, 2010; Triga et al., 2023), and it is considered to be an opportunistic bacteria with low or no pathogenicity to aquatic animals (Mohamad et al., 2019). The sequence MZ901309, within the Gigantis clade, shows the highest identity to *V. gigantis* (99.6% identity) and *V. coralliirubri* (99.6% identity). Therefore, most of the pathogenicity in *S. foetidus* is expected to come from vibrios with OK036808, OK037006, and OK037007 sequences, respectively related to *V. owensii*, *V. fortis*, *V. gigantis*, three sequences that were collectively detected as the dominant Vibrio in 6 of the 9 diseased individuals sampled (Supplementary Table S1). Additionally, it cannot be discarded that a longer version of sequence MZ901309, present in a 7th diseased individual, may end resulting conspecific with *V. gigantis*. The fact that all these putatively pathogenic strains were not dominant in the agar plates obtained for all 9 diseased individuals suggests that vibriosis is likely a secondary infection that aggravates the course of disease but not the primary etiological agent. Nevertheless, further research based on metagenomics of diseased individuals sampled at several stages of disease would be required to definitively resolve this question.

**Figure 7:**
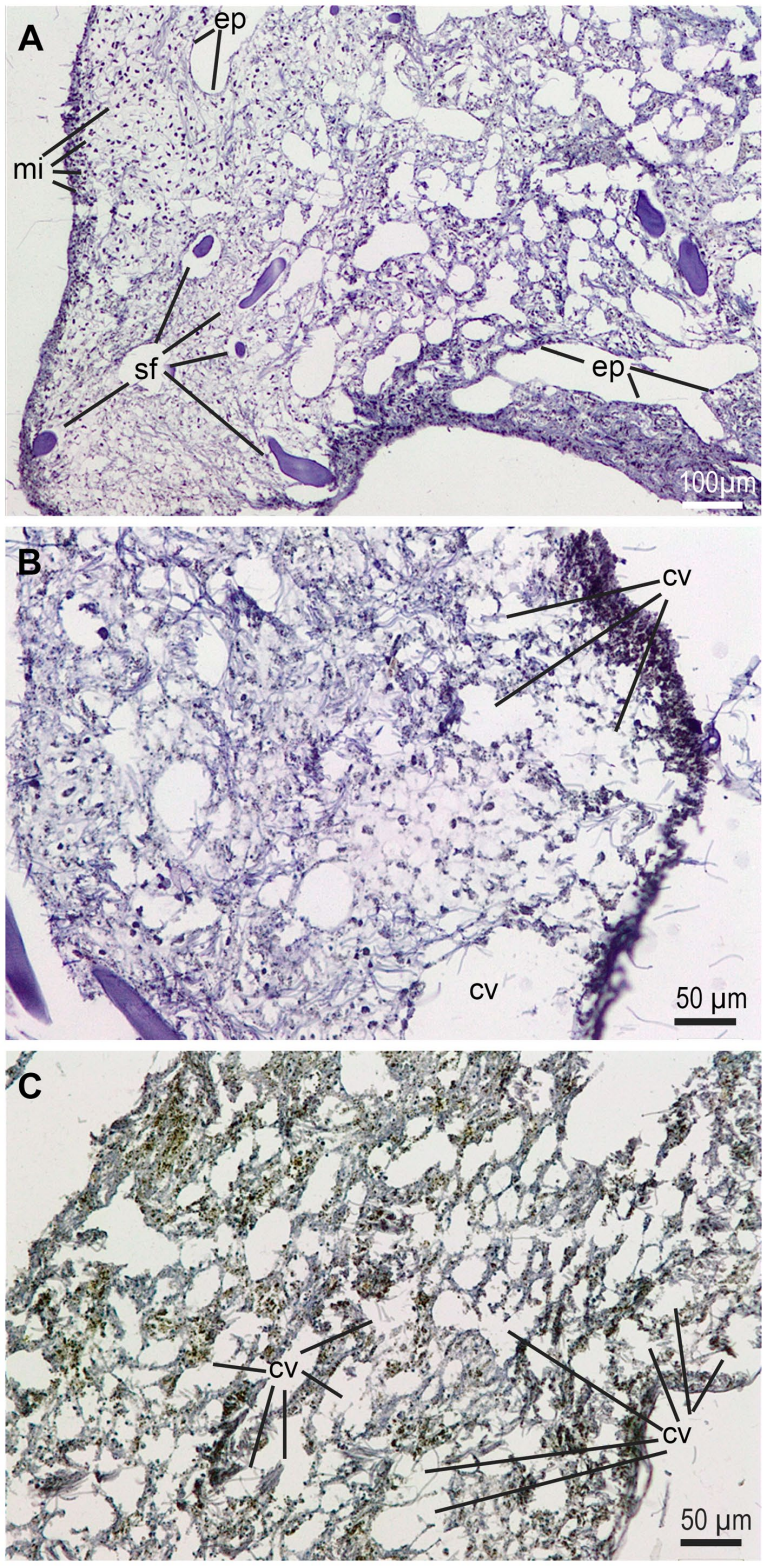
Histological views of the mesohyl of healthy and diseased regions in a symptomatic individual at early stage of disease. **(A)** Healthy tissue area showing regular abundance of subdermal microbes (mi), typically bacteria and cyanobacteria, along with a properly structured choanosome with aquiferous channels provided of the endopinacotelium (ep) and spongin fibers (sf). (**B, C**) Disease areas of choanosome where the mesohyl is being “digested” and large cavities (cv) appear. Note that those cavities do not have the proper epithelium of the aquiferous channels.

Although light microscopy is a low resolution approach to learn about the histological consequences of bacterial infections in sponges, a comparative examination of diseased (i.e., partially necrotic) and macroscopically asymptomatic choanosome zones reveals larger abundance of microbes (i.e., a richer natural microbiome) in healthy tissue areas, as well as important tissue alterations in diseased areas, involving destruction of aquiferous channels and choanocyte chambers after partial digestion (likely with cytolysis and degradation of intercellular collagen fibrils) of the mesohyl (Figure 7). Obvious damage to the spongin fibers was not detected, but transmission electron microscopy would have been necessary to definitively exclude it.

### 3.3 Historical temperature record

**Figure 8:**
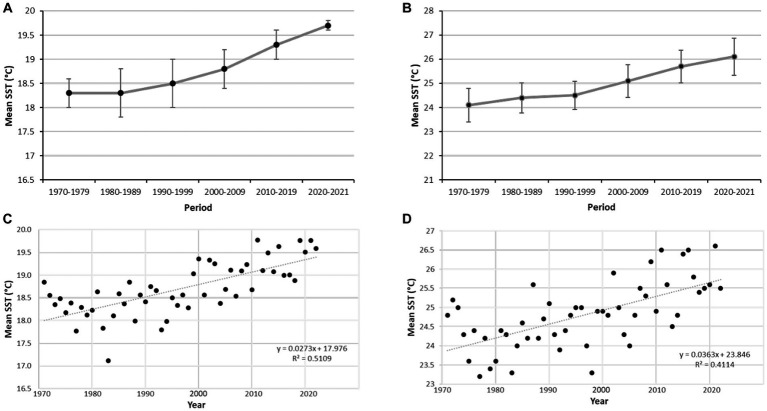
Sea surface temperature (SST) values recorded at Izmir station (see Figure 1) for 50 years. **(A)** Values of annual SST averaged (±SD) for decadal periods. **(B)** Mean values of SST in September averaged (±SD) for decadal periods. **(C)** Annual mean values of SST. **(D)** Mean values of SST in September plotted by years.

The SST data reveal a consistent, progressive increase of the mean temperature of superficial seawater over the last 50 years (Figure 8), irrespective of the data being treated as decadal or annual values. More important, the analysis of 2021 data reveals that the mean temperature was higher than 24°C through June (24.9°C), July (27.1°C), August (27.5°C), and September (25.5°C). The maximum mean SST for 2021 raised as high as 28.5°C in August.

## 4 Discussion

### 4.1 Sponge vibriosis

Events of mass mortality in Mediterranean benthic communities have been more and more often associated with episodes of water warming (reviews in: Cerrano et al., 2000; Garrabou et al., 2009; Antoniadou et al., 2023). However, it has proven very difficult to identify the ultimate mechanisms through which water warming leads to disease and death. Our results suggest that long periods of elevated seawater temperatures facilitate infection by thermo-dependent pathogenic microbes, as indicated by proliferation in the tissue the sponge *Sarcotragus foetidus* of several species of *Vibrio* at a moment when water temperature reached the highest value in the 50 years history of local registers. However, our approach cannot decide whether the proliferation of the identified *Vibrio* spp. is the initial origin of the sponge disease or those *Vibrio* spp. opportunistically colonize sponges that were already diseased. In any case, three of the detected Vibrios (OK036808, OK037006, and OK037007) are either strains or undescribed species closely related to *V. owensii* (clade 1), *V. fortis* (clade 3), and *V. gigantis* (clade 5), respectively. These three species are known to be pathogenic for a wide array of marine organisms (Cano-Gómez et al., 2010; Ushijima et al., 2012; Wang et al., 2016; Liu et al., 2018, 2021; Triga et al., 2023), which may now also include sponges. Therefore, even if the *Vibrio* spp. isolated from the tissue of *S. foetidus* are not the initial cause of disease but secondary opportunistic agents, the pathogenicity of some of them will aggravate the course of the disease. To our knowledge, evidence suggesting that *Vibrio* may be involved in sponge disease has only been obtained for *Ircinia variabilis*, the diseased tissue of which led to laboratory growth of colonies of *Vibrio rotiferianus* (Stabili et al., 2012). Since the discovery that species of *Vibrio* were the causative agent of coral bleaching over 20 years ago (Kushmaro et al., 1997, 2001; Ben-Haim and Rosenberg, 2002; Ben-Haim et al., 2003a), these bacteria have been blamed for mass mortalities in various groups of benthic invertebrates worldwide, particularly cnidarians and molluscs.

The necrotic lesions observed at the external surface and the peripheral mesohyl of diseased individuals of *S. foetidus* (Figure 7) are similar to those previously described from intermediate disease stages in *I. fasciculata*, with initially small round lesions progressing to larger necrotic subepithelial wounds that later became large ulcerous cavities in the mesohyl, a process documented by transmission electron microscopy (Maldonado et al., 2010a). The genus *Vibrio* is long known to display important collagenolytic and chitinolytic activities, which are related to their pathogenicity (Smith and Merkel, 1982; Li and Roseman, 2004). The extracellular enzymes of *Vibrio*, which include collagenases and chitinases, have been associated with the lysis of cnidarian tissues (Sussman et al., 2008; Vezzulli et al., 2010) and may also be behind the extensive mesohyl destruction noticed in several species of the genus *Ircinia* and in *S. foetidus*, since the intercellular medium of the mesohyl is extremely rich in collagen fibrils. Attack to the spongin fibers of keratose sponges, which are essentially collagenous structures that may incorporate chitin (Ehrlich et al., 2007), is also described in reports of other several keratose species (Vacelet et al., 1994b). Attack to fibers has not been noticed in our histopathological study of *S. foetidus*. Interestingly, a majority (but not all) of reports in the literature about sponge diseases involve demosponges with spongin skeleton (i.e., “corneous demosponges”) rather than demosponges with a skeleton made of silica parts. Therefore, the hypothesis that corneous demosponges may be more susceptible to epidemic diseases than their siliceous counterparts deserves further examination. The diversity and abundance of bacteria and archaea in the mesohyl of many sponge species is typically high, particularly in the so-called “high microbial abundance (HMA)” sponges (Gloeckner et al., 2014). This is also de case of most corneous demosponges in the family Irciniidae (Sarà et al., 1973; Vacelet, 1975; Vacelet and Donadey, 1977; Stabili et al., 2012) to which *S. foetidus* also belongs. Whether being an HMA sponge is an added condition that favours microbial diseases is another issue that deserves further examination. In this sense, future approaches to sponge diseases would better be complemented with electron microscopy – which was not possible in the present study – in order to characterize in greater detail cellular and ultrastructural aspects of infections and their lesions.

Vibrios are an important component of the coastal bacterioplankton, with up to 10^5^ cell mL^−1^ in the warm season (Leyton and Riquelme, 2008). Thus, at some point, they may also serve as food to many sponges, which largely rely on bacterioplankton as main food source (Maldonado et al., 2012). An infection from the inhalant channels of the sponges during filter feeding is a pathway that, for the time being, cannot be ruled out. Note that translocation of living microbes from the inhalant channels to the mesohyl has been documented in feeding experiments with the sponge *Hymeniacidon perlevis* (Maldonado et al., 2010b). There is abundant literature indicating that *Vibrio* spp. are more abundant in warm waters, particularly proliferating above 17°C (Thompson et al., 2004). In the Mediterranean Sea, proliferation during warm SST periods has been demonstrated for various species of *Vibrio* (*V. harveyi*, *V. splendidus*, and *V. coralliilyticus*) that caused massive mortality in the Mediterranean gorgonian *Paramuricea clavata* (Vezzulli et al., 2010). It has also been suggested for some coral-killing species of *Vibrio* (*V. parahaemolyticus*, *V. coralliilyticus*, *V. shiloi*) that these pathogens would proliferate seasonally from reservoirs in the sediments or in other benthic hosts where they overwinter (Sussman et al., 2003; Ben-Haim et al., 2003b). Urged by the unforeseen disease outbreak and without specific funding for this research topic, we were unable to follow the evolution of the *Vibrio* populations in the seawater of the sponge habitat over time, but such a sampling may help to clarify initial steps of the infection in future studies. Some non-pathogenic species of *Vibrio* are known to occur in regular symbiosis with many invertebrates including sponges, since these bacteria have a wide repertoire of enzymes that allow them to perform an array of catabolic functions and to use a varied array of carbon and nitrogen sources (Thompson and Polz, 2006). However, the *Vibrio* species detected in diseased individuals of *S. foetidus* do not appear to be symbionts, since they were not retrieved in cultures inoculated with tissue of asymptomatic individuals, neither in summer nor in winter.

Our present results for *S. foetidus*, along with a previous detection of vibriosis in the sponge *I. variabilis* (Stabili et al., 2012), provide a small body of evidence suggesting that vibriosis is a likely pathway through which seawater warming would lead to sponge disease and eventually to mortality, particularly in keratose sponges. We postulate that if future cases of sponge disease in temperate and tropical areas are approached from this perspective, more cases of vibriosis are likely to emerge as either direct etiological agents or as the result of secondary infections that aggravate disease outcome.

### 4.2 Taxonomic interpretation of 16S rRNA Vibrio sequences

To decide a specific taxonomic adscription for the 16S rRNA sequences of *Vibrio* identified in the sponges is not easy. Since the beginning of the 1990’s, the 16S rRNA marker has been established as a primary tool for bacterial taxonomic identification and it is widely accepted that values lower than 98.7% in 16S rRNA sequence similarity typically indicate different species (Kim et al., 2014). However, as the numbers of new sequences continues to grow in some bacterial genera, the 16S rRNA markers loses discriminating power and this might be the case of *Vibrio*. The new emerging view is that taxonomic identifications based solely on the 16S rRNA need to be treated with caution, since lack of informative content and variations in its evolutionary rate can cause deviations from the “98.7% similarity threshold” (Yarza et al., 2014). Because of these problems, newly isolated bacterial strains have begun to be taxonomically identified using whole genome data, through a variety of genome similarity indexes (Chun and Rainey, 2014). However, the 16S rRNA is still used to confirm these genomic assignments, because, unlike genome data, 16S rRNA sequences are available for almost every bacterial species known (Chun et al., 2018).

Some of the *Vibrio* strains newly isolated in the present study show a 16S rRNA pairwise identity higher than 98.7% with different well-accepted species. For example, *Vibrio* sp. OK037007 shows a 99.7% similarity with *V. gigantis*, a 99.6% with *V. coralliirubri*, a 99.5% with *V. artabrorum*, and 98.8% with *V. toranzoniae*, suggesting that the marker has a very low-resolution power for this group of closely related species. Therefore, although the use of the 16S rRNA remains as one of the most practical approaches to tentatively identify newly isolated bacterial strains, genomic and phenotypic information (both, when possible) would be necessary to support formal species descriptions (Rosselló-Móra and Amann, 2015). In the absence of genomic data, we have interpreted the retrieved sequences in terms of relatedness to existing *Vibrio* species, but avoiding to propose new taxa for those 16S rRNA sequences with identities lower than 98.7% to accepted *Vibrio* spp.

### 4.3 Ecological impact

*Sarcotragus foetidus* has been reported from different zones around Turkey: Sea of Marmara (Ostroumoff, 1896), Aegean Sea (Kocataş, 1978; Ergüven et al., 1988; Çinar and Ergen, 1998; Çinar et al., 2002) and Levantine coasts (Evcen and Çinar, 2012). Due to its large size and abundance, *S. foetidus* is predicted to play an important functional role in the benthic communities where it is abundant, through its filter-feeding activity, nutrient recycling, and by its body providing refuge and suitable habitat for many invertebrates (Pavloudi et al., 2016; Çinar et al., 2019). Because of its large size, the species is also prone to be easily impacted by trawling, netting, ghost fishing, and alike fishing activities (Gerovasileiou et al., 2018). However, despite the potential relevance of this sponge species in ecology conservation of the sublittoral benthic communities, its basic biology and actual effectives remain poorly known for every Turkish population.

Our estimates of natural sponge density are in agreement with figures in other studies. Voultsiadou et al. (2008) noted that density of *S. foetidus* can be up to 5 individuals m^−2^ in Eastern Mediterranean communities, and Enrichetti et al. (2020) reported up to 7.7 individuals m^−2^ of this species in the North Western Mediterranean Sea. Our results on body size distribution are also in agreement with a previous study performed in Ligurian Sea (Enrichetti et al., 2020). Regarding the incidence of disease in the sponge population, the values detected for *S. foetidus* are within the range of those reported in a variety of other sponge diseases from different locations. For instance, the “sponge necrosis syndrome,” described in *Callyspongia* aff. *biru* from the Indian Ocean had a 30–33% incidence (Sweet et al., 2015). In the massive sponge mortality at the Adriatic Sea in 2009, Di Camillo et al. (2013) determined that the percentage of incidence varied across species, being 88% for *Sarcotragus spinosulus*, 10% for *I. variabilis*, and only 2% for *Spongia officinalis*. In the disease event that affected *I. fasciculata* at the Mediterranean coasts of Spain in 2008–2009, a 57% of the individuals were found to be affected in populations from southern Spain (Maldonado et al., 2010a) and between 80 and 100% in populations of eastern Spain (Cebrian et al., 2011). In contrast, some phylogenetically close species, such as *I. variabilis*, *Ircinia oros*, and *Sarcotragus spinulosum* were minimally affected in those 2 years. The mortality rate of *S. foetidus* – in the narrow time window of our study – was conservatively estimated at 9%, since we were unable to determine how many of the partially necrotizing individuals (i.e., 27% of population) would die. Although this incidence appears to be moderate, we hypothesize that the studied populations of *S. foetidus* will be recurrently infected in subsequent summers. The bulk of the population of *S. foetidus* is concentrated in the upper sublittoral (depths of only 1–7 m), a shallow bathymetric range that increases the chances of this species to develop future *Vibrio*-related diseases facilitated by the summer seawater warming resulting from the typical summer stratification of the water column in the Mediterranean sublittoral (Coma et al., 2009).

In 2021, monthly mean SST values were higher than 24°C for 4 consecutive months (June, July, August and September) at the study zone of the Aegean Sea, with a maximum mean SST as high as 28.9°C in August 2021. This maximum value is in line with maxima of 28°C and 30°C reached in the Adriatic Sea in 2009, which unchained a severe mass mortality of sponges, cnidarians, and bryozoans (Kružić et al., 2016). According to the 50 years linear trend in the increase in SST at the study area (Figure 8), similarly high SST values are expected nearly every future summer. If so, even a moderate increase in annual values of mortality will result in *S. foetidus* being eliminated from the upper sublittoral in few years, modifying the current bathymetric range of the species. The large size and the high actual abundance of this sponge (up to 4 ind. m^−2^) indicates that it is not a trivial member of the community regarding functionality (i.e., refuge provision to microfauna, removal of bacterioplankton, release-uptake of N and P inorganic nutrients that are crucial for primary production, etc.). Thus, a rapid species disappearance from the upper sublittoral may unchain associated ecological effects in the benthic community. Alternatively, sponges might develop mechanisms to fight back vibriosis in a future scenario of progressive ocean warming, since putative anti-*Vibrio* spp. defences have been reported for some demosponges (Raharja et al., 2019). Midterm monitoring programs would be required to track the prevalence of epidemic disease in some coastal areas of the Eastern and Western Mediterranean and to assess the effects of mass sponge mortalities on both the population effectives and the functional ecology of benthic communities.

## 5 Conclusion

Events of invertebrate mass mortality are often associated with global warming of the oceans. However, for some invertebrate groups, it has proven very difficult to identify the processes/mechanisms through which water warming causes disease and, ultimately, death. This uncertainty applies in particular to keratose sponges, a group of demosponges abundant in shallow water that is characterized by lacking siliceous skeletons and is particularly susceptible to epidemic-like mass mortality. Our study detects proliferation of several species of *Vibrio* (thermo-dependent and culturable bacteria) in the keratose demosponge *Sarcotragus foetidus* during a mass mortality episode associated to a heat wave in the Aegean Sea (Eastern Mediterranean) in 2021. These results come into agreement with a previous 2012 study that also detected growth of *Vibrio* during an epidemic mortality of the also keratose demosponge *Ircinia variabilis* in the Western Mediterranean. Therefore, our study relaunches the view that vibriosis is a likely process through which heat waves lead to sponge disease and eventual mortality, particularly in keratose demosponges. It is hoped that the findings and their interpretation will inspire further research to better understand sponge diseases and their ecological implications.

## Data availability statement

The datasets presented in this study can be found in online repositories. The names of the repository/repositories and accession number(s) can be found in the article/Supplementary Material.

## Ethics statement

The manuscript presents research on animals that do not require ethical approval for their study.

## Author contributions

ED: Conceptualization, Formal analysis, Methodology, Writing – original draft, Investigation. FÖ: Investigation, Methodology, Supervision, Writing – review & editing. LL: Methodology, Writing – review & editing, Software. MM: Methodology, Conceptualization, Formal analysis, Funding acquisition, Supervision, Writing – original draft.

## Footnotes

^1^http://tree.bio.ed.ac.uk/software/figtree/
