# Supplementary material for: Mass mortality of the keratose sponge Sarcotragus foetidus in the Aegean Sea (Eastern Mediterranean) correlates with proliferation of Vibrio bacteria in the tissues
Source: Front Microbiol. 2023 Dec 1;14:1272733. doi: 10.3389/fmicb.2023.1272733 (PMC10722426; doi:10.3389/fmicb.2023.1272733)
Supplement: Supplementary file 1 [file Table_1.DOCX]

**Supplementary Table S1.** Dominant Vibrio sequence isolated from each of the diseased individuals of *Sarcotragus foetidus* sampled for the study.

| **Diseased**  **individual** | **Vibrio sequence**  **(accesion number)** |
| --- | --- |
| Individual 1 | OK036808 |
| Individual 2 | OK036808 |
| Individual 3 | MZ900920 |
| Individual 4 | OK037006 |
| Individual 5 | OK037006 |
| Individual 6 | OK037006 |
| Individual 7 | OK036959 |
| Individual 8 | OK037007 |
| Individual 9 | MZ901309 |
