# Supplementary figures and images for: Mass mortality of the keratose sponge Sarcotragus foetidus in the Aegean Sea (Eastern Mediterranean) correlates with proliferation of Vibrio bacteria in the tissues
Source: Front Microbiol. 2023 Dec 1;14:1272733. doi: 10.3389/fmicb.2023.1272733 (PMC10722426; doi:10.3389/fmicb.2023.1272733)

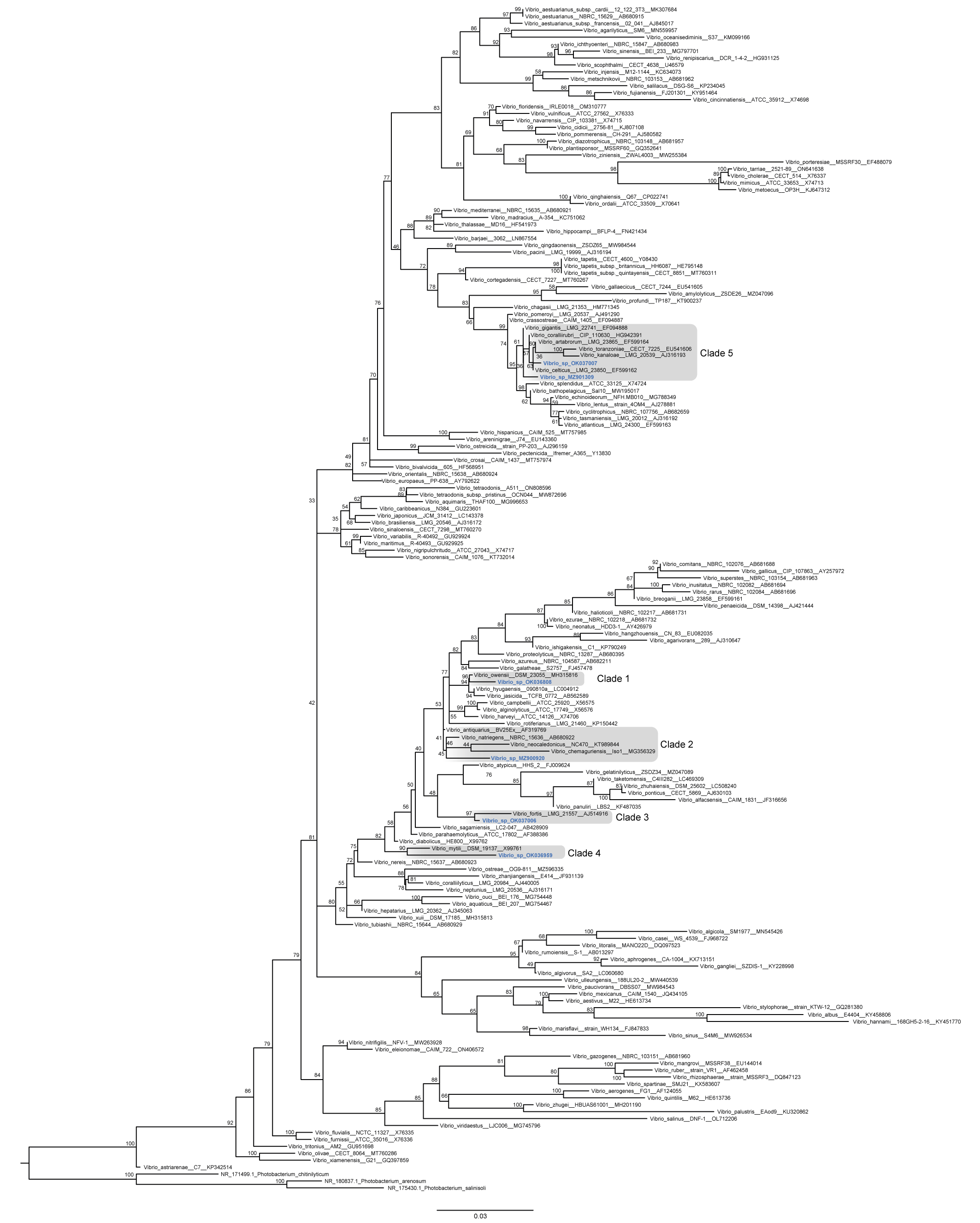

Supplement: Supplementary file 4 [file Image_1.TIF]
